# The pericyte connectome: spatial precision of neurovascular coupling is driven by selective connectivity maps of pericytes and endothelial cells and is disrupted in diabetes

**DOI:** 10.1038/s41421-020-0180-0

**Published:** 2020-06-16

**Authors:** Tamas Kovacs-Oller, Elena Ivanova, Paola Bianchimano, Botir T. Sagdullaev

**Affiliations:** 1 Burke Neurological Institute, White Plains, NY 10605 USA; 2 Szentagothai Research Centre, University of Pécs, Pécs, H-7624 Hungary; 3 Department of Ophthalmology, Weill Cornell Medicine, New York, NY 10065 USA

**Keywords:** Mechanisms of disease, Calcium signalling

## Abstract

Functional hyperemia, or the matching of blood flow with activity, directs oxygen and nutrients to regionally firing neurons. The mechanisms responsible for this spatial accuracy remain unclear but are critical for brain function and establish the diagnostic resolution of BOLD-fMRI. Here, we described a mosaic of pericytes, the vasomotor capillary cells in the living retina. We then tested whether this net of pericytes and surrounding neuroglia predicted a connectivity map in response to sensory stimuli. Surprisingly, we found that these connections were not only selective across cell types, but also highly asymmetric spatially. First, pericytes connected predominantly to other neighboring pericytes and endothelial cells, and less to arteriolar smooth muscle cells, and not to surrounding neurons or glia. Second, focal, but not global stimulation evoked a directional vasomotor response by strengthening connections along the feeding vascular branch. This activity required local NO signaling and occurred by means of direct coupling via gap junctions. By contrast, bath application of NO or diabetes, a common microvascular pathology, not only weakened the vascular signaling but also abolished its directionality. We conclude that the exclusivity of neurovascular interactions may thus establish spatial accuracy of blood delivery with the precision of the neuronal receptive field size, and is disrupted early in diabetes.

## Introduction

Local changes in neural activity evoke a vascular response that is spatially restricted to the activated region. The ubiquitous nature of the microvasculature and the diversity of routes through which blood can be distributed make this task challenging. Furthermore, blood perfusion is shifted to accommodate for changing activity patterns. To accomplish this, functional hyperemia is thought to comprise a series of events with a spatial precision that enables discrimination of the active site from its resting neighbor: (1) sensing local changes, (2) transmission of vasoactive signals along the irrigating vascular branch, culminating in (3) vasomotor response that directs blood to the active region. The mechanisms responsible for this spatial accuracy are not clear, but are critical for brain function and establish the diagnostic power and precision of BOLD-fMRI^1^.

The strategic location of capillaries within synaptic layers where neurotransmitters are released supports their role as both sensors and responders to neural activity^2,3^. Here, pericytes, the only contractile cells along the vast capillary network may fulfill both of these roles. Contrary to long-held beliefs, pericytes express smooth muscle actin^4^, which enables capillary diameter changes in response to electrical, pharmacological, and sensory stimuli^2,5–7^. In light of these observations, it is unclear why the vasomotor response to stimulus is first observed in larger vascular branches, away from the sites of neural activity and not at proximal capillary regions^6,8^. While further verification is needed, this intriguing spatial segregation is consistent with a role of larger precapillary regions in the blood supply^9,10^. More importantly, on a temporal scale, it suggests the existence of a presently unknown signaling mechanism that enables the rapid propagation of a vasoactive signal from the capillary region towards the feeding vascular branch^11^. Direct gap junction (GJ)-mediated communication among pericytes and other vascular elements plays a key role in vasomotor response propagation^6,12,13^. Indeed, in the isolated retina vasculature, electrotonic pulses propagate radially along the network of pericytes and endothelial cells (ECs)^14^. However, this radial spread is inconsistent with the directional nature of the vasomotor response occurring in the living brain. Following focal stimulation, the vasomotor response tends to propagate stronger upstream of the irrigating vascular branch, a phenomenon observed in multiple systems, including retina^9^, olfactory bulb^15^, cerebral cortex^16^, and skeletal muscles^17^. These studies raise the following key questions: (1) what is the nature of cell-to-cell interactions between the different regions of the vascular tree that mediate the spatio-temporal precision of a vasomotor response, (2) how do changes in local neural activity induce directional bias within a radially coupled vascular syncytium, and (3) how does vascular signaling accommodate for changes in sensory modalities?

Recent characterization of the “vascular relay” in the retina, a specialized vascular region along a capillary branch with distinct distribution of Cx43-containing GJs across ECs and pericytes, established the structural foundation for vascular cell connectivity^13^. Here, in retina wholemounts, a self-contained brain structure with a defined vasculature, we experimentally tested the hypothesis that the spatial accuracy of vasomotor response is driven by a precise and discriminatory connectivity map among vascular cells — pericytes and ECs. We further probed whether, and ultimately how, these connectivity maps dynamically shifted to accommodate for changing sensory input. To accomplish this, we used a combination of light simulation, direct cell-to-cell tracer coupling, multiphoton imaging of calcium activity, and the measurements of vasomotor response. The utility of an intact retina wholemount preparation has allowed us to rigorously combine natural light stimulus with selective pharmacological tools to study fundamental properties of vascular signal transmission with high temporal and spatial resolution. We then used this model system along with capillary blood flow measurements in live animals to determine how the neurovascular impairment may contribute to diabetic retinopathy, a common vascular pathology in diabetic patients^18,19^.

## Results

### Pericytes form a mosaic in the retina microvasculature

**Fig. 1:**
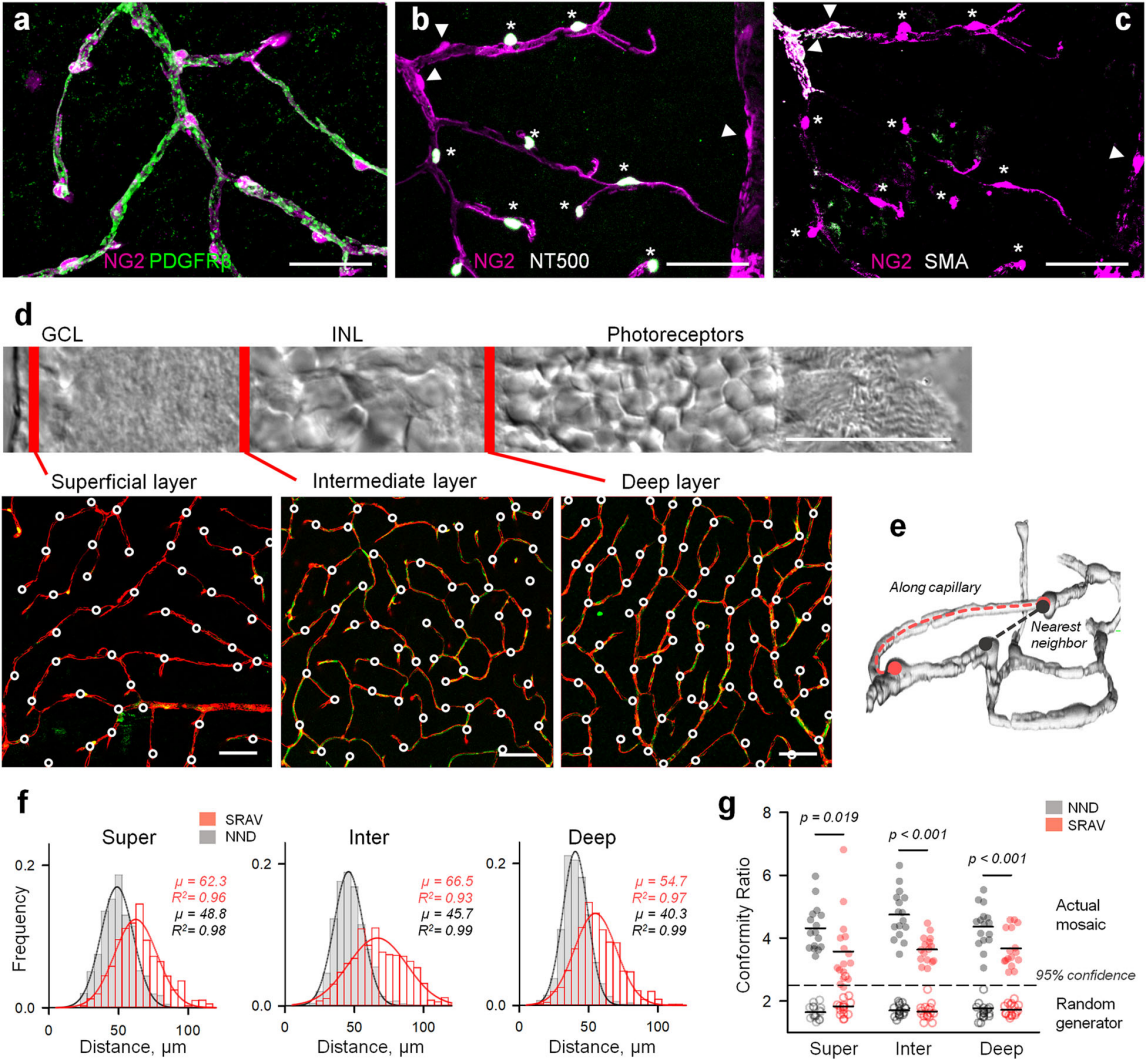
Mosaic or pericytes in the retinal vasculature. **a**–**c** Pericytes (asterisks) were distinguished from smooth muscle and transitional mural cells (arrowhead) using a selective uptake of NT500. **d** Distribution of pericytes across three retinal vascular layers. For visualization and follow-up analysis, each circle was placed over the pericyte cell body. Locations of the vascular layers are marked by red in a transmitted image of a retinal cross-section. GCL and INL are ganglion and inner nuclear layers, respectively. All scales are 50 µm. **e** Illustration showing how both the inter-pericyte SRAV and the NND were measured. **f** Distance frequency histograms in each vascular layer. Mean distances (*µ*) and the Gaussian goodness of fit (*R*^*2*^) for each distribution are indicated to the right. **g** Conformity Ratios (mean/SD) for NND and SRAV. Dotted line represents a 95% confidence threshold over randomly generated values drawn from the same distribution. Each point in the plot represents a mean value within the individual animal (*n* = 16 mice) with a combined number of measures *n* = 3647. *P*-values are from one-way ANOVA with Tukey’s post-hoc test.

Functional hyperemia is thought to rely on coordinated activity across a broad vascular network, directing blood to active regions. Since a vasomotor response will depend on placement of the vasoactive elements, the knowledge of pericyte distribution across a broad capillary network is needed. In NG2-DsRed mice, all vessels, including arterioles, veins, and capillaries, were readily identified by fluorescently labeled mural cells. In these mice, we combined a set of anatomical features with a selective uptake of NeuroTrace 500/525 (NT500) assay to unambiguously distinguish pericytes from smooth muscle cells (SMCs) (Fig. 1a–c)^19,20^. In all three vascular layers within a 300 × 300 μm retina patch, pericyte cell bodies were notated (Fig. 1d). Two sets of measurements were calculated: the shortest route along the vessel (SRAV), and the nearest-neighbor distance (NND) between pericytes, regardless of their capillary residence (Fig. 1e). Across all vascular layers, we found that pericytes were distributed regularly (Gaussian goodness of fit R^2^ = 0.93–0.99, *n* = 16 mice). This regularity was maintained even in the superficial vascular layer (*R*^2^ = 0.93), despite areas occupied by large precapillary regions (Fig. 1f). Overall, inter-pericyte distances decreased from superficial to deep vascular layer, consistent with increased capillary density. As an exception, SRAV distance was longest in the intermediate layer, likely due to the inherent tortuosity of the capillary network. To test for pericyte regularity, we used the conformity ratio (CR), the ratio of the mean distance to its standard deviation^21^. We found that CRs were significantly above either Ready-Reckoner thresholds (2.3 for *n* = 50 samples)^22^ or randomly generated values, drawn from the same data set (SRAV: 3.63 ± 0.76 vs. 1.74 ± 0.27, *P* < 0.001; NND: 4.47 ± 0.72 vs. 1.71 ± 0.19, *P* < 0.001, paired *t*-test, *n* = 48 ROIs in *n* = 16 mice for each, Fig. 1g). Notably, the CRs for NND were higher compared to SRAV (superficial: 4.31 ± 0.72 vs. 3.58 ± 1.13, *P* = 0.019, intermediate: 4.75 ± 0.77 vs. 3.64 ± 0.42, *P* < 0.001; deep: 4.36 ± 0.64 vs. 3.67 ± 0.59, *P* < 0.001 paired *t*-test, *n* = 16 mice for each), suggesting that the pericyte mosaic was not simply driven by direct pericyte-to-pericyte connectivity along the same vascular branch, an evidence that their placement may be optimized for volume regularity across the retina.

### Vascular cells establish discriminatory GJ connections with each other but not with neurons or glia

What is the mechanism of vasomotor response propagation along the vascular tree? The answer to this question will depend not only on spatial distribution of vasoactive elements but also on the nature and the strength of the underlying cell-to-cell interaction. Direct and rapid coupling among neurovascular elements via GJs is a key event in vasomotor response propagation; GJ block completely abolished response propagation, but not its initiation^6^. To establish a neurovascular connectivity map, or “neurovascular connectome” of the retina, we used single-cell injection of GJ-permeable probe Neurobiotin (NB) and traced all cells that were coupled to the injected cell^23^. We chose NB for the assessment of GJ-mediated cellular connectivity due to its established utility in a variety of tissues^24^, including retinal vascular cells^12,13^. In this study, we have injected cells within the superficial layer for the following reasons: (1) a robust access to all cell types for both tracing and targeted stimulation, without causing physical damage to the wholemount tissue and thus substantially reducing the possibility for non-specific tracer pickup, (2) all blood vessel types, from the arteriole to finest capillaries, are present in the superficial layer, allowing for the coupling assessment along a contiguous vascular tree, and (3) diverse cell types are present, including astroglia, that are known to play an active role in neurovascular coupling^7^. Nevertheless, the NB coupling was assessed across all vascular layers. To identify each cell type, we relied on a set of characteristic anatomical features, such as cellular morphology and stratification depth (Supplementary Figs. S1 and S2). These features were then further confirmed using molecular and immunohistochemical markers.

**Fig. 2:**
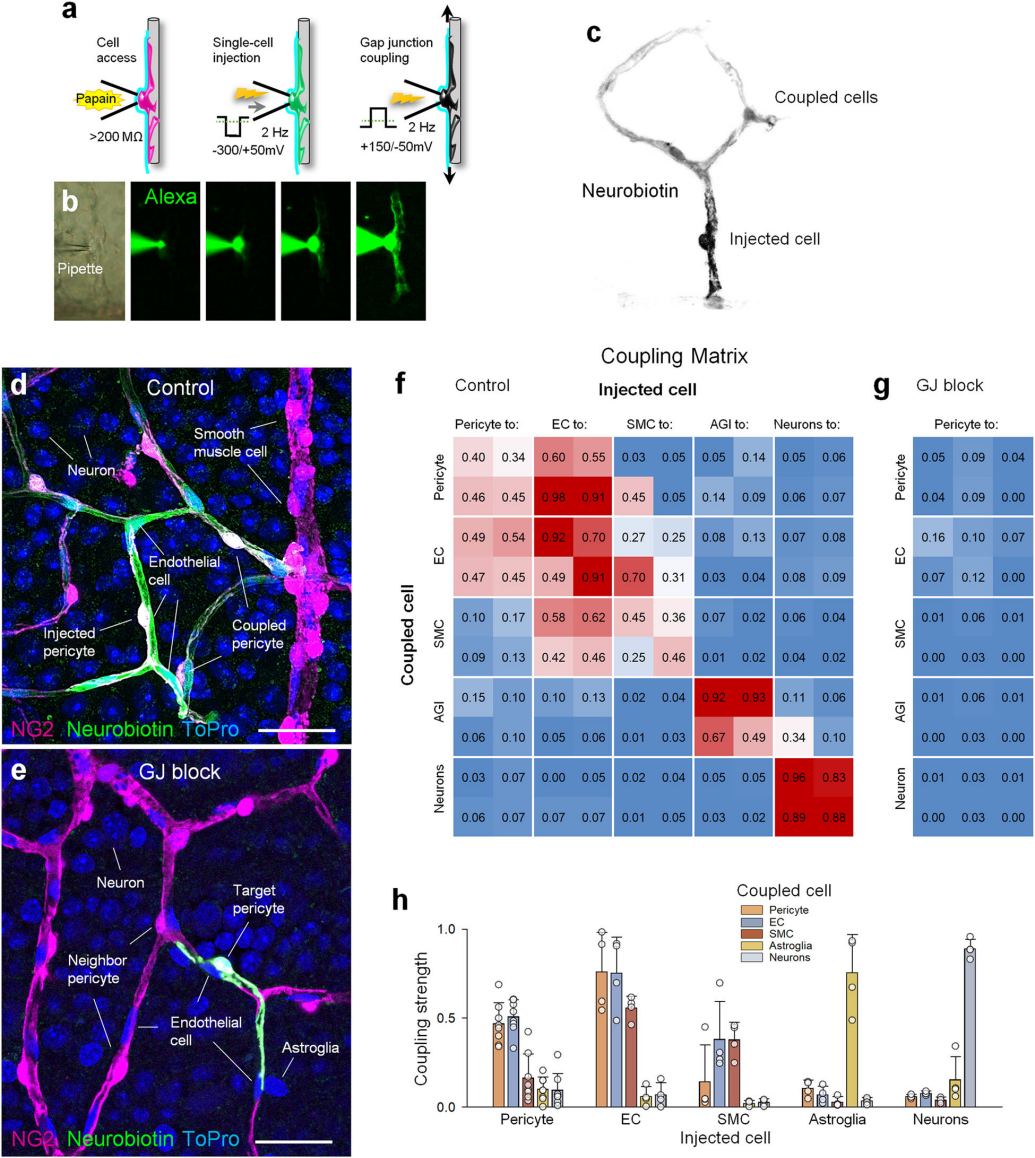
Coupling connectome of the neuro-glia-vascular unit. **a** Illustration of the experimental design to target and trace GJ-mediated connections of an identified cell using a combination of probes with different size/permeability. **b** With a patch-clamp pipette, large Alexa488 dye and small NB tracer are co-injected into the target cell. Using this assay, distinct cells can be selectively targeted and their connections traced (Supplementary Figs. S1 and S2). **c** Appearance of NB spread from the targeted cell is revealed with an anti-streptavidin antibody. **d** Confocal reconstruction of the tracer spread across GJ-coupled cells in the retinal wholemount. **e** Treatment with a GJ blocker (MFA, 40 µM) precludes tracer spread. Scale bar, 25 µm. **f, g** Cell-type-specific connectivity matrices. In this heatmap matrix, injected cell type is shown on top, with its coupled neighbor down. Numbers in each box are average CS values between the target cell and its six neighbors within individual animals (representative values, not full dataset from all animals are shown). Higher value and a warmer color indicate stronger coupling. **h** Summary of connectivity strength for all individual pairs across *n* = 8 mice injected in pericytes and *n* = 5 mice per each other cell target condition.

As illustrated in Fig. 2a–c, 15 min following NB injection, a “chain” of GJ-coupled cells could be revealed (Supplementary Video S1). To unambiguously identify the injected cell, we supplemented the NB-containing intracellular solution with a larger, GJ-impermeable Alexa dye (Fig. 2b–d). Furthermore, the NB spread was precluded by meclofenamate (MFA, 40 µM), a GJ blocker (Fig. 2e, g). Together, these confirm that NB was selectively infused into a targeted cell and its spread occurred through GJs, and not by nonspecific NB uptake.

Next, for each cell type, coupling strength (CS), a measure of NB spread (see Materials and methods) was calculated to yield a coupling matrix of the retinal neurovascular unit (Fig. 2f, g). The analysis of coupling matrices revealed a number of insights. The majority of connections were homotypic and highly discriminatory across cell types; vascular cells coupled to other vascular cells, astroglia to astroglia, and neurons to neurons. Here, due to a lack of heterotypic GJ coupling, we simplified the approach and treated all neuronal cells as a single group, without further discrimination. Pericytes were coupled predominantly to other neighboring pericytes (CS = 0.47 ± 0.11) and ECs (0.51 ± 0.09, *P* = 0.24) and significantly less to arteriolar SMCs (0.16 ± 0.13, *P* < 0.001), glia and neurons (0.10 ± 0.06 and 0.10 ± 0.09, respectively, *P* = 0.001, multi-comparisons ANOVA, Tukey post-hoc, *n* = 8 mice for each pair). Interestingly, SMCs on the arterioles were among the least coupled cells (Fig. 2f, h; Supplementary Fig. S1), evidence for a mechanism that restricts the changes in blood supply to the activated region and thus improves spatial accuracy of functional hyperemia.

### Pericyte and EC connectivity maps shift in response to changing sensory input

**Fig. 3:**
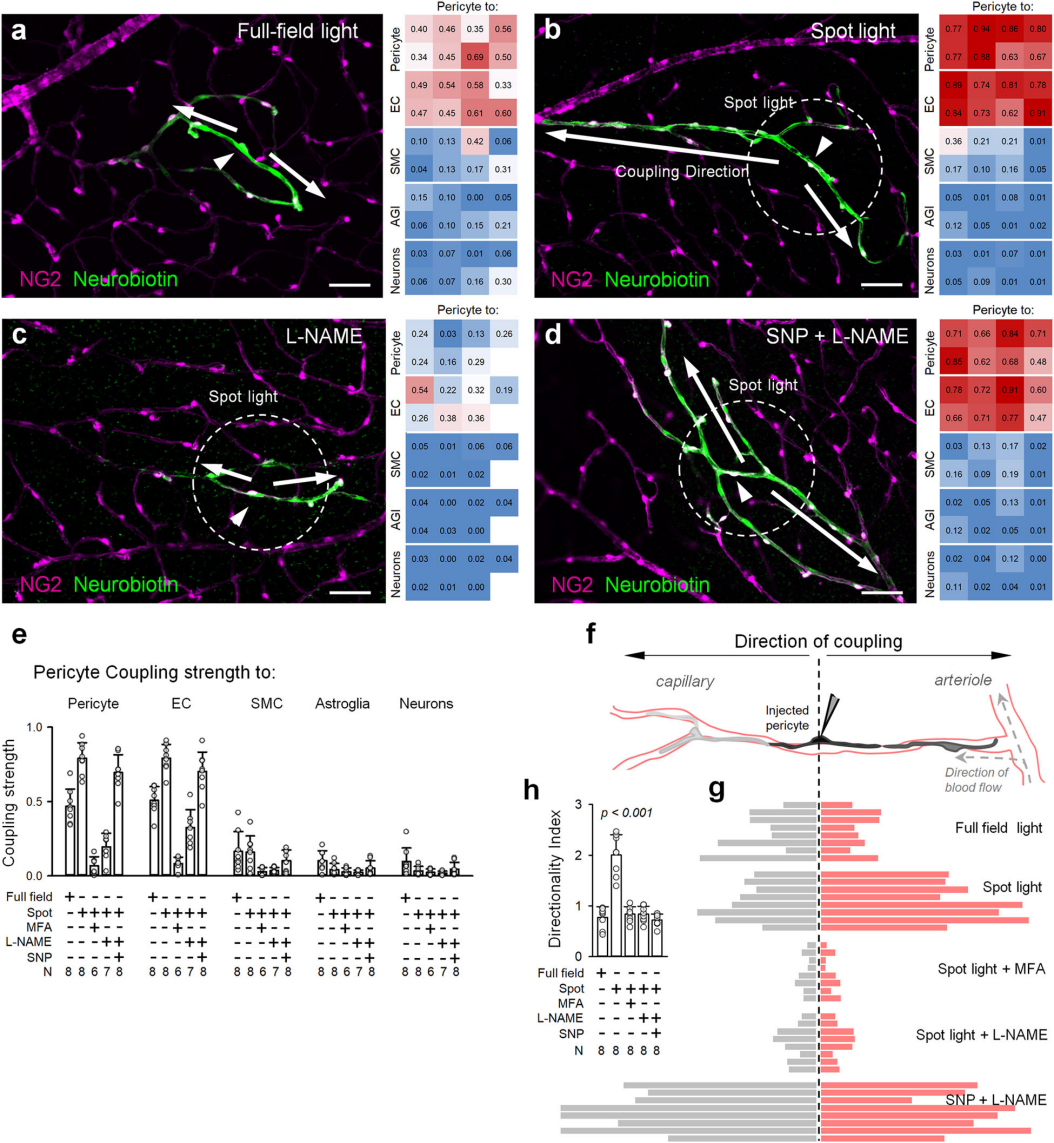
Strength and directionality of vascular coupling are driven by focal sensory input and is mediated by NO signaling. **a** Spatially broad sensory stimulation (full-field light) produces weak pericyte-to-pericyte and pericyte-to-EC coupling. **b** Focal sensory stimulation under capillary area induces strong vascular coupling along the capillary branch, biased toward the feeding arteriole. **c** Blockade of NO signaling abolished increased CS induced by a focal sensory stimulation (150 μm light spot). **d** Exogenous application of NO donor significantly increases CS but fails to restore its directionality. Scale bars, 50 µm. **e** Summary of pericyte cell CS under each experimental condition. **f** Experimental design and assessment of vascular cell coupling directionality. **g** Vascular cell CS up- and downstream from the injected pericyte (dashed vertical line). Each bar pair (left and right) is from individual mice under each experimental condition (*n* = 8 mice per each group). **h** Summary of pericyte cell coupling directionality under experimental conditions.

Having established a spatial mosaic of pericytes and their exclusive connectivity maps, we next used the living retina preparation and its natural stimulus light, to test how these interactions change with sensory input. In the brain, neurons are tuned to preferential modalities. In particular, retinal neurons respond strongest to stimuli within their receptive fields (RFs). We hypothesized that this activity would translate to the spatial extent of vascular activation. If true, this would establish a theoretical resolution limit for BOLD-fMRI at the range of 50–200 μm, typically found for neuronal RFs. To accomplish this, we compared vascular cell coupling under two experimental conditions: (1) full-field flickering light (4 Hz) and (2) spot flickering light (4 Hz, 150 µm diameter, that approximates the excitatory center of a retinal ganglion cell receptive field)^25,26^. To ensure that equal amounts of light were delivered under both approaches, a stationary background was maintained at a mean value of full-field stimulus around a flickering spot center. For easier access and parfocal view of diverse vascular branches, pericytes in the superficial layer were targeted, while NB spread was evaluated across all vascular layers. For consistency across multiple samples, we injected pericytes 3–4 branch points away from the feeding artery (~300 µm, Fig. 3a–d), thus allowing sufficient space in both up- and downstream directions of the vascular tree. In addition to the CS, we also measured its directionality index (DI), the ratio of upstream vs. downstream vascular cell coupling bias (Fig. 3f, Materials and methods). As such, DI values >1 indicate connectivity bias toward the feeding artery. We found that under a full-field light stimulus, pericytes were coupled to other pericytes and ECs with CS = 0.47 ± 0.12 and 0.51 ± 0.09 (*n* = 8 mice each, Fig. 3e), respectively. No directional bias was evident (DI = 0.77 ± 0.21), consistent with an even distribution of the pericytes. In stark contrast, spot light stimulus significantly increased CS over full-field stimulation (CS = 0.79 ± 0.11, *P* < 0.001, ANOVA with Tukey’s post-hoc, *n* = 8 mice, Fig. 3b). Surprisingly, this was not driven by a symmetric increase in coupling in both directions from the injected pericyte, as would be predicted from their structural regularity (Fig. 3f–h). Instead, focal sensory input strengthened connections along the shortest path in the direction of the feeding branch (DI = 2.0 ± 0.4 vs. 0.77 ± 0.21 in full-filed, *P* < 0.001, ANOVA with Tukey’s post-hoc, *n* = 8 mice). Again, both coupling and directionality were blocked by MFA (40 µM, CS = 0.07 ± 0.06; DI = 0.84 ± 0.15, *n* = 6 mice).

### Directionality of vasomotor response is driven by local NO signaling

Our findings provide direct evidence for a dynamic connectivity map utilizing a structurally rigid vascular framework^27^, and suggest its role in spatial accuracy of functional hyperemia. To further test this, we determined whether the increase in vascular cell coupling was indeed driven by neural activity. Synthesis of NO is a key event in the neurovascular coupling^5,7^. In contrast to other signaling molecules like arachidonic metabolites and K^+^ that are predominantly released by the glia, NO is released by neurons and acts directly on vasculature^28–30^. We, therefore, hypothesized that blocking nitric oxide synthase (NOS), an enzyme responsible for activity-induced NO production would diminish vascular cell coupling. Consistently, during spot light stimulation, application of L-NAME (100 µM), a broad-spectrum NOS inhibitor, significantly reduced vascular cell coupling compared to spot light alone (0.20 ± 0.09 vs. 0.79 ± 0.11, *P* < 0.001, ANOVA with Tukey’s post-hoc, *n* = 7 and eight mice, respectively). It also abolished directionality of vascular cell coupling (Fig. 3f–h, 0.84 ± 0.16 vs. 2.0 ± 0.4, *P* < 0.001, ANOVA with Tukey’s post-hoc, *n* = 7 and 8 mice, respectively). In the presence of L-NAME, vascular cell coupling was also significantly lower than under full-field sensory stimulation (0.2 ± 0.09 vs. 0.47 ± 0.12, *P* < 0.001, ANOVA with Tukey’s post-hoc, *n* = 7 and 8 mice, respectively), suggesting sustained coupling under suboptimal sensory stimulation. However, this activity was not sufficient to evoke a directional response. Was this strengthened directionality of vascular cell coupling driven by simply an overall increase in NO production due to optimized neuronal stimulation, or did it rather require a spatially defined local increase in NO? To address this, we next measured the magnitude of NB spread in response to spot light stimulation in the presence of both L-NAME, to block local NO production, and bath applied sodium nitroprusside (SNP, 100 µM). Bath application of NO significantly increased the CS (Fig. 3g, CS = 0.7 ± 0.12 vs. 0.2 ± 0.09 in L-NAME alone, *P* < 0.001, ANOVA with Tukey’s post-hoc, *n* = 8 and 7 mice, respectively). However, this was not accompanied by the characteristic directional profile of the NB coupling in response to a spatially restricted stimulus (spot DI = 2.0 ± 0.4 vs. bath SNP DI = 0.72 ± 0.12, *P* < 0.001, ANOVA with Tukey’s post-hoc, *n* = 8 mice each), suggesting that local NO production was necessary for directional coupling.

**Fig. 4:**
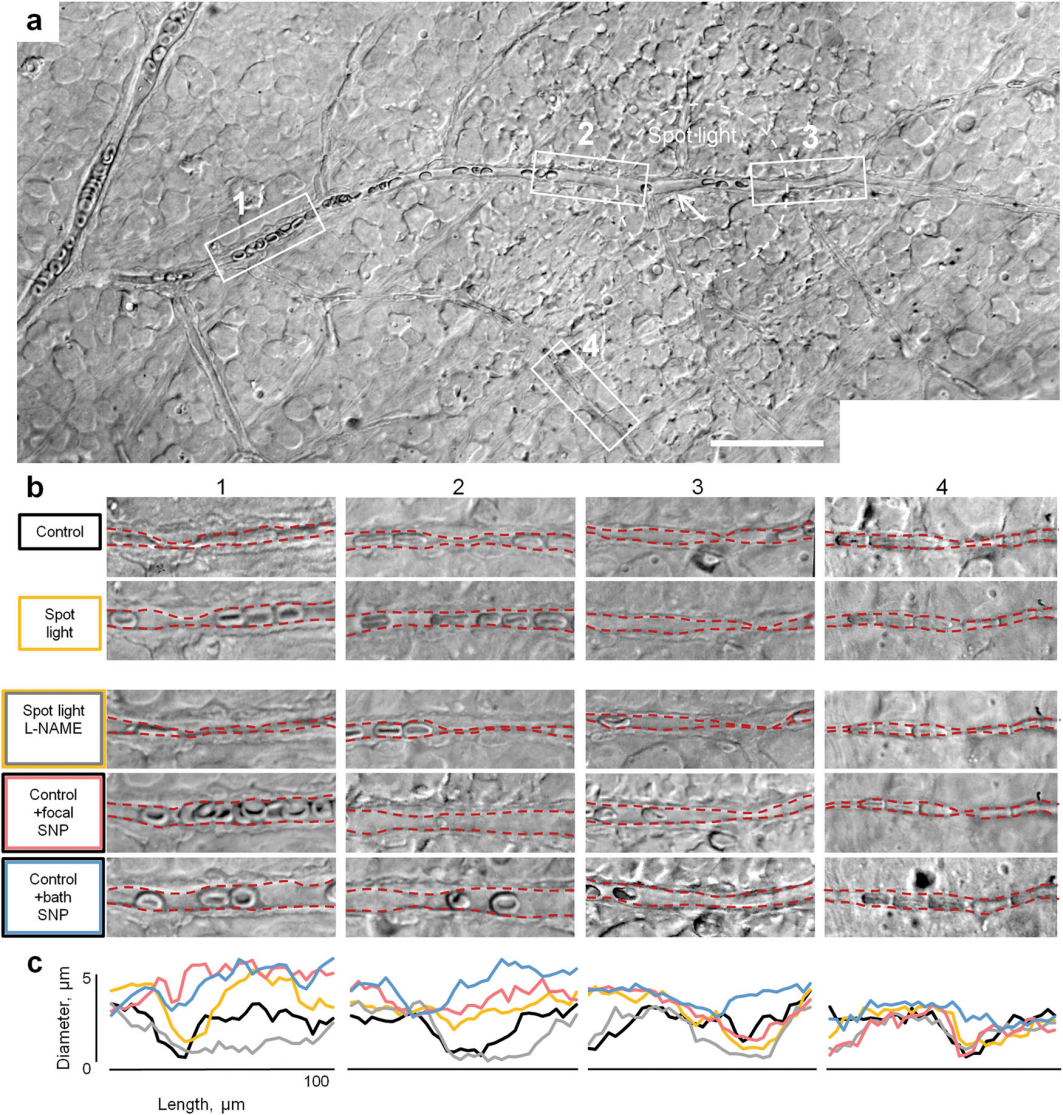
Directionality of light-induced vasodilation is driven by local, but not global NO signaling. **a** In the retina wholemount, vascular diameter changes were monitored at 100 µm long ROIs (boxed) below, above the site of stimulation and at the collateral branch (arrow). **b** Magnified ROIs under the indicated experimental conditions. **c** Volumetric analysis of vascular diameter changes at each ROI. Largest vasodilation was observed in the direction toward the feeding branch (ROIs 2 and 1) in response to either flickering spot of light (150 µm, 4 Hz), or focal SNP puff (100 µM), but not during global light stimulation or in the presence of L-NAME (100 µM).

Next, in freshly dissected retina wholemounts, we found that spot light stimulation resulted in capillary dilation that propagated upstream along the feeding vascular branch, but neither downstream nor collateral regions (Fig. 4a, b). This is consistent with an earlier established GJ-mediated coupling map (Fig. 3g). We then tested whether locally applied NO was sufficient for this directional vasomotor response. Under full-field illumination, we used a patch pipette to puff SNP around an individual pericyte. To reduce the spatial spread of SNP, the pipette was positioned downstream to the vascular branch relative to perfusion flow. Similar to a spot light flicker, focal SNP produced directional vasomotor activity (Fig. 4; note dilation in ROIs 1 and 2, but not 3 or 4). This directional response was lost during consequent bath application of SNP, resulting in broad vascular dilation in all vascular branches (Fig. 4b, lower panels). While further validation is needed, these data indicate that spatially accurate stimulation is responsible for directionality of both cellular connectivity and the vasomotor response propagation^31^.

### Timing of Ca^2+^ change and vasomotor response is contractile cell type specific

**Fig. 5:**
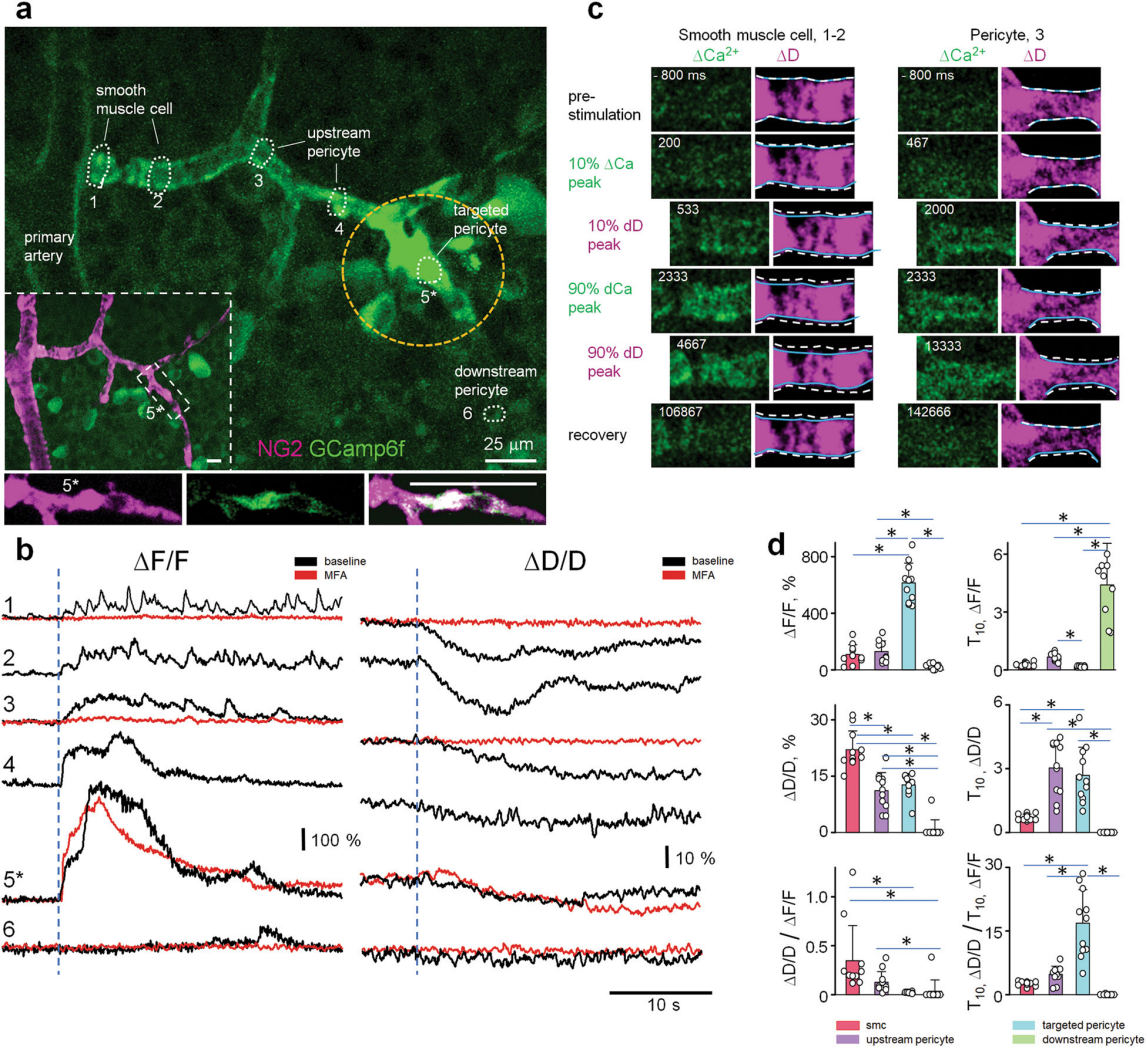
Vasomotor response is shaped by contractile cell-type-specific temporal dynamics between Ca increase and vasomotor response. **a** Directional calcium increase in a vascular branch after local electric stimulation of a capillary pericyte (5) in an NG2-DsRed-GCamp6f mouse. Inability to distinguish the targeted cell is due to GCaMP activation in surrounding cells (dashed circle). The bottom panels show targeted pericyte (5*). **b** Temporal kinetics of calcium rise and vasomotor response in contractile cells along the vascular branch shown in **a** (baseline, black trace). Calcium rise in non-targeted contractile cells and propagation of the vasomotor response was blocked by 40 mM MFA (red); they were not affected in the targeted pericyte. **c** Remote SMCs at the artery have faster calcium rise and stronger vasomotor response than pericytes adjacent to the stimulation site. Pre-stimulation outlines of the blood vessels are shown by a dashed line in all frames, while current outlines are marked by solid blue lines. **d** Calcium rise, vasomotor response, and Excitation-Constriction Coupling is significantly different between pericytes and SMCs. Data are shown as means ± SD; 11 samples, 6 mice, one-way ANOVA. *P < 0.05.

Cellular basis of the vasomotor response dynamics remains controversial. This is in part due to a limited ability to control the activation site and timing in vivo^8,9^. To overcome these limitations, we used freshly dissected retina wholemounts from NG2-Cre-GCamp6f mice. While in the earlier study, tamoxifen induction of Cre resulted in targeted expression of GCamp6f in mural cells exclusively^8^, in our mice, constitutive activity of the NG2 promoter resulted in GCamp6f expression in all retinal cells, including neurons, glia, and vasculature. Next, we crossed our NG2-Cre-GCamp6f-EGFP mice with an NG2-DsRed line to label vasculature. Ubiquitous expression of GCamp6f (green fluorescence) and restricted expression of DsRed in the mural cells (magenta fluorescence) enabled us to simultaneously assess Ca^2+^ dynamics in the entire neurovascular unit and conduct a rigorous volumetric analysis of vasomotor response. To achieve precise timing and mimic local neuronal activation, individual pericytes were directly depolarized by a patch electrode. In NG2-Cre-GCamp6f-DsRed retina, we stimulated a capillary pericyte (2 ms, 10 µA) while imaging GCamp6f and the vascular diameter under a two-photon microscope (Fig. 5a, location 5). Short electric stimulation resulted in pericyte activation, followed by local transient activation of Muller cells (dashed circle marks the limit of the Muller cell activation) and propagation of Ca^2+^ wave through the vascular branch. DsRed-expressing contractile cells along the vascular branch were identified (labels in Fig. 5a) and temporal calcium dynamics with vasoconstriction were measured at these locations at 15 frames per second (Fig. 5b). The Ca^2+^ increase was almost instantaneous in the vascular branch leading towards the supplying artery (locations 1–4), but did not propagate much toward the vein (location 6). In spite of simultaneous Ca^2+^ increase, vasoconstriction, initiated by Ca^2+^, was significantly faster and stronger in SMCs (location 1–2) in comparison to pericytes (location 3–4). Again, propagation of both Ca^2+^ wave and vasomotor response relied on vascular GJs and was abolished by 40 µM MFA (shown by the red line in Fig. 5b, locations 1 and 3) without affecting Ca^2+^ rise and vasoconstriction at the targeted pericyte (location 5). To quantify temporal kinetics of the Ca^2+^ wave and vasomotor response as well as their relation to each other, we used the following parameters: peak amplitudes of Ca^2+^ increase and vasomotor response (∆*F*max/*F* and ∆*D*max/*D*), time from stimulation to 10% and 90% of Ca^2+^ and vasoconstriction response. Figure 5c shows corresponding time frames for both smooth muscles and a pericyte. Both Ca^2+^ increase and vasomotor response were delayed in the pericyte in comparison with the SMCs.

Finally, for each contractile cell type, we determined the temporal relationship between Ca^2+^ increase and vasomotor response (Fig. 5d). Ca^2+^ increase (∆*F*/*F*) in the targeted pericyte was significantly higher than that in the other contractile cells (615% ± 139% in targeted pericyte; 129% ± 75% in upstream pericyte; 109% ± 68% in SMC; 27% ± 17% in downstream pericytes; 11 branches, 6 mice). Ca^2+^ increase in upstream pericytes was also significantly higher than in downstream pericytes. In the SMCs, Ca^2+^ increase tended to be lower than in pericytes. However, the peak vasomotor response (∆*D*/*D*), initiated by the Ca^2+^ wave was significantly larger in SMCs (22% ± 5% in SMC; 11% ± 4% in upstream pericyte; 12% ± 3% in the targeted pericyte; 1% ± 1% in downstream pericyte; 11 branches, 6 mice). There was little or no vasoconstriction in downstream pericytes. The efficiency of constriction, defined as the ratio of constriction to Ca^2+^ increase (∆*D*/*D*/∆*F*/*F*), was significantly higher in SMCs (0.35 ± 0.3 in SMC; 0.13 ± 0.11 in upstream pericytes; 0.02 ± 0.01 in the targeted pericyte; 0.03 ± 0.1 in downstream pericytes; 11 branches, 6 mice). Temporal kinetics were also different between pericytes and SMCs. Time of 10% Ca^2+^ increase (T10∆*F*/*F*) in the downstream pericytes, if detected, was significantly delayed (285 ± 90 ms in SMC; 661 ± 194 ms in upstream pericytes; 164 ± 35 ms in the targeted pericyte; 4400 ± 2162 ms in downstream pericytes; 11 branches, 6 mice). Surprisingly, when the time of vasoconstriction was compared (T10∆*D*/*D*), it was significantly shorter in the remote SMCs than in the upstream pericytes adjacent to the targeted pericyte (706 ± 150 ms in SMC; 3030 ± 1283 ms in upstream pericytes; 2958 ± 1399 ms in the targeted pericyte; 11 branches, 6 mice). When we adjusted time of vasoconstriction to the time of Ca^2+^ increase (T10∆*D*/*D*/T10∆*F*/*F*), SMCs were still significantly faster (2.6 ± 0.5 in SMC; 4.8 ± 1.9 in upstream pericytes; 19 ± 9 in targeted the pericyte; 11 branches, 6 mice). Thus, higher ∆*D*/*D*/∆*F*/*F* coupling efficiency in SMCs compared to pericytes may explain the earlier observations of faster response kinetics of vasomotor response in arteriolar branches during capillary stimulation.

### Diabetic retinopathy disrupts vascular connectivity map, impairing directionality and extent of vasomotor response

**Fig. 6:**
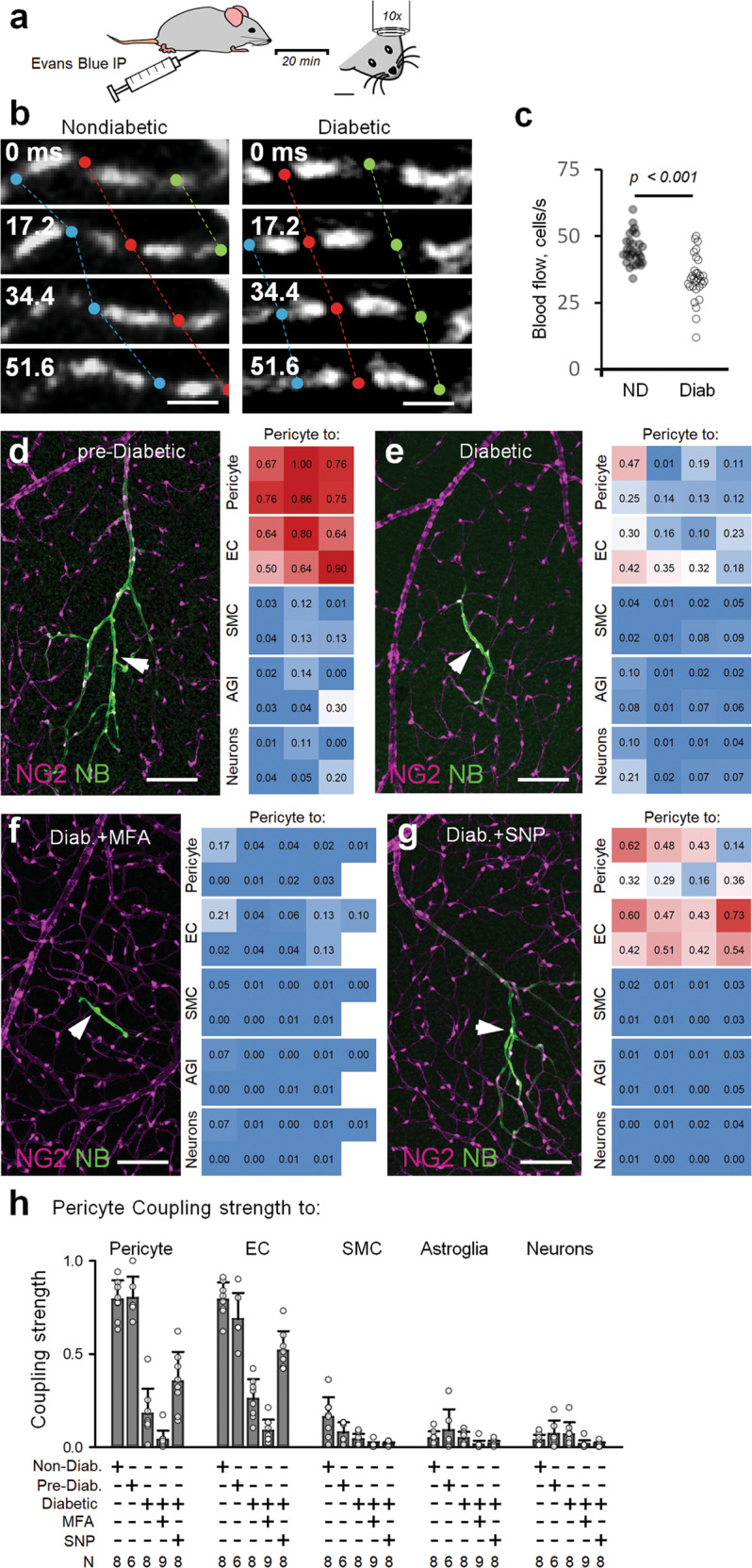
Functional connectivity maps are disrupted in diabetes. **a** Experimental paradigm of measuring retinal capillary blood flow in mice in vivo. **b**, **c** Assessment of retinal capillary blood flow in vivo reveals decline in blood flow in the diabetic mice. **d**, **e** Evaluation of cell coupling following pericyte injection ex vivo retinal wholemount at the onset of diabetes (pre-diabetic, 2–3 weeks post STZ treatment) (**d**) and during established diabetes (3–4 months post STZ treatment) (**e**). **f** In diabetic conditions, the GJ-mediated vascular cell coupling is further blocked in the presence of MFA (40 µM). **g** Effect of NO donor (SNP, 100 µM) on GJ-mediated vascular cell coupling in diabetic conditions (3–4 months post STZ treatment). **h** Summary of experimental data under each condition (*n* = 6–9 animals per group).

In this study, we have demonstrated that the vascular cell coupling, propagation of Ca^2+^ increase and the resultant vasomotor response rely on active GJs. We also show that these interactions are strengthened by and dynamically shifted with changing sensory modality. In diabetic retinopathy (DR), GJs between ECs and possibly ECs and pericytes are selectively diminished^6,27^. We next used the streptozotocin (STZ)-induced Type 1 diabetes model to determine how selective elimination of GJs in the vascular relay affected cellular interactions and vasomotor response during the progression of DR (see Materials and methods). We studied two time points: pre-diabetic and diabetic. Pre-diabetic animals were 2–3 weeks post last STZ injection and their non-fasting glucose was below 250 mg/dL. Diabetic animals were 3–4 months after the last STZ injection with persistent glucose levels >300 mg/dL. First, to validate an STZ-induced animal model, we tested for the evidence of impaired capillary blood flow in the retina in vivo (Fig. 6a and Supplementary Videos S2 and S3)^32^, an early pathology in patients with diabetic retinopathy^33,34^. Three to four months after STZ injection, the baseline blood flow was reduced significantly in diabetic animals compared to non-diabetic placebo animals (Fig. 6b, c; non-diabetic 44.8 ± 5.7 cells/s vs. diabetic 34.2 ± 8.4 cells/s; *t*-test, *P* < 0.001; six capillaries per animal, five mice per group).

**Fig. 7:**
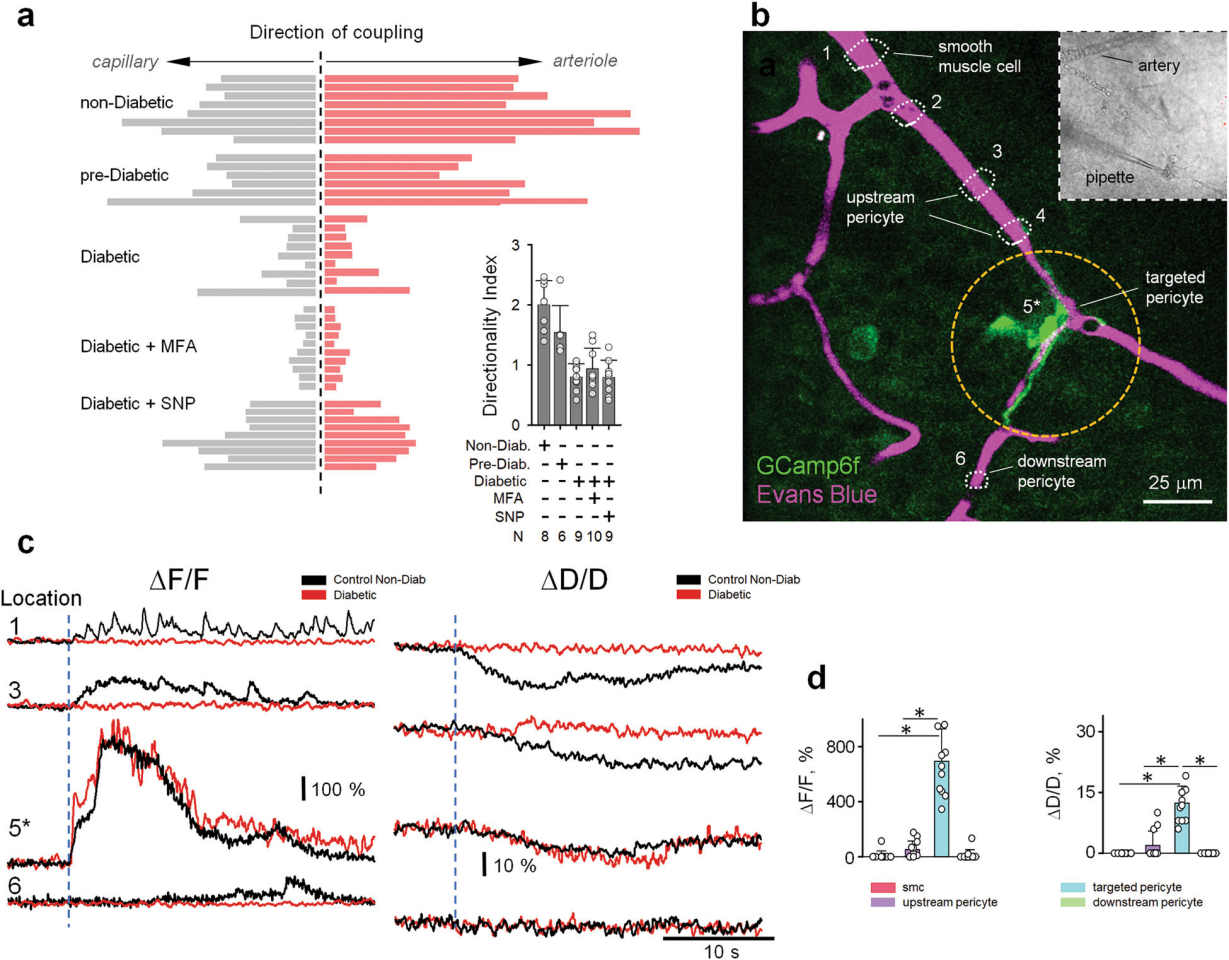
Vascular coupling, Ca^2+^ signaling and vasomotor response are disrupted in diabetes. **a** In the diabetic retina, the directionality of vascular cell coupling was abolished. Global NO application enhanced cellular coupling but did not restore its directionality. **b** In living retinal wholemount of NG2-GCamp6f mouse, local depolarization of a pericyte by an electrode lead to local calcium rise and vasoconstriction. **c** Temporal kinetics of calcium rise and vasomotor response in contractile cells along the vascular branch shown in **a** (Diabetic, red traces) in comparison with responses of control, Non-Diabetic animal from Fig. 5b (black traces). **d** Local Ca^2+^ and vasomotor responses did not propagate along the vascular branch in diabetic (red trace) in contrast to responses from control non-diabetic animals from Fig. 5b (black traces). Data are shown as means ± SD; 12 samples, 6 mice, one-way ANOVA. *P < 0.05.

Next, in freshly dissected retina wholemounts we determined whether changes in blood flow were paralleled by a reduction in GJ-mediated vascular cell coupling. As shown in Fig. 6, CS of both pericyte-pericyte (P-P) and pericyte-ECs (P-E) in diabetic mice was diminished compared to both non-diabetic (CS for P-P: 0.18 ± 0.14 vs. 0.79 ± 0.11, *P* < 0.001, and CS for P-E: 0.25 ± 0.11 vs. 0.79 ± 0.09, *P* < 0.001, ANOVA with Tukey’s post-hoc, *n* = 8 mice in each group) and pre-diabetic animals (CS for P-P: 0.18 ± 0.14 vs. 0.80 ± 0.12, *P* < 0.001, and CS for P-E: 0.25 ± 0.11 vs. 0.69 ± 0.14, *P* < 0.001, ANOVA with Tukey’s post-hoc, *n* = 8 and 6 mice, respectively). Similarly, the directionality of vascular cell coupling was reduced in diabetic retina (DI for non-diabetic = 2.0 ± 0.4, pre-diabetic = 1.54 ± 0.45 and diabetic = 0.8 ± 0.21; *P* < 0.001, ANOVA with Tukey’s post-hoc, *n* = 6–9 mice in each group). A pharmacological block of GJs eliminated all the remaining coupling (Fig. 6f, CS = 0.04 ± 0.05 and DI = 0.94 ± 0.34 in 40 µM MFA), suggesting the progressive nature of the GJ-mediated communication in diabetes. Interestingly, while the application of SNP increased the overall CS in diabetic retina, it failed to reverse the coupling directionality (Fig. 7a), suggesting a more nuanced regulatory mechanism of vascular vasomotor response propagation.

Next, in the living retina wholemount, we focally stimulated a pericyte with a patch pipette while monitoring Ca^2+^ dynamics and vasomotor response (Fig. 7b). To visualize blood vessel diameter, the mouse was intraperitoneally injected with Evans Blue to label plasma albumin and, therefore, blood (Fig. 7b, magenta)^32^. Evans Blue was contained inside the blood vessel and the outlines of the blood vessel were clearly visible. Thus, the lumen of the blood vessels could be reliably measured at the early stage of the diabetic retinopathy^6^. Contractile cells were identified in transmitted light based on their characteristic “bump on the log” shape (Fig. 7b). Stimulation of a targeted pericyte (location 5) induced Ca^2+^ increase, which did not propagate beyond the stimulated cell (Fig. 7b, c). In line with the restricted Ca^2+^ wave, vasoconstriction was limited to the blood vessel underneath the stimulated pericyte. Both Ca^2+^ wave and vasoconstriction at the targeted pericyte was similar between wild-type (WT) and diabetic retinas (Fig. 7d, WT: targeted pericyte ∆*F*/*F* = 615% ± 139%, ∆*D*/*D* = 12% ± 3%, *n* = 11, 6 mice; diabetic: targeted pericyte ∆*F*/*F* = 693% ± 234%, ∆*D*/*D* = 12% ± 4%, *n* = 12, 6 mice; ∆*F*/*FP* = 0.35, ∆*D*/*DP* = 0.84, *t*-test). In contrast to WT, propagation of the calcium wave and vasoconstriction was significantly different in diabetic retinas (WT: upstream pericyte ∆*F*/*F* = 129% ± 75%, ∆*D*/*D* = 11% ± 5%, *n* = 11, 6 mice; diabetic: upstream pericyte: ∆*F*/*F* = 52% ± 62%, ∆*D*/*D* = 2% ± 4%, *n* = 12, 6 mice; ∆*F*/*FP* = 0.013, ∆*D*/*DP* < 0.001, *t*-test; WT: SMCs ∆*F*/*F* = 109% ± 68%, ∆*D*/*D* = 22% ± 5%, *n* = 11, 6 mice; diabetic: SMCs ∆*F*/*F* = 9% ± 33%, ∆*D*/*D* = 0 ± 0%, *n* = 12, 6 mice; ∆*F*/*FP* < 0.001, ∆*D*/*DP* < 0.001, *t*-test). The absence of propagation in the downstream direction was similar in WT and diabetic retinas (WT: downstream pericyte ∆*F*/*F* = 29% ± 16%, ∆*D*/*D* = 1% ± 3%, *n* = 11, 6 mice; diabetic: downstream pericyte: ∆*F*/*F* = 13% ± 38%, ∆*D*/*D* = 0 ± 0%, *n* = 12, 6 mice; ∆*F*/*FP* = 0.22, ∆*D*/*DP* = 0.31, *t*-test). Thus, diabetic retinopathy caused disruption of communication among the vascular cells similar to the effect of GJ blocker in WT.

## Discussion

In this work, we reveal a functional connectivity map of pericytes and ECs that mediate the spatial and temporal precision of neurovascular signaling in the retina. We found that pericytes formed a precise 3D mosaic across the retina. However, in response to sensory stimuli, the vasomotor activity propagated asymmetrically along the feeding branch. This directionality was observed when the stimulus matched a neuronal receptive field center size and was driven along a highly discriminatory GJ-mediated relay between pericytes and ECs. Pericytes and ECs connected predominantly to other upstream neighboring pericytes and ECs, and less to arteriolar smooth muscles, and not to surrounding neurons and glia. Below, we discuss the implications of our findings, potential mechanisms and new questions that may arise.

### GJ-mediated connectome of the neurovascular unit

Functional hyperemia is thought to rely on the coordinated activity across a broad vascular network — sensing activity changes and then directing blood to an active region. With the exception of a few recent reports^8,35^, the mechanistic studies of spatial interactions during functional hyperemia have mainly focused on correlations between neural activity and vasomotor elements within a restricted region. Here, in the living retina, we mapped pericytes across a broad capillary network to reveal that they form a mosaic. Interestingly, the observed mosaic was not simply among the pericytes on the same vascular branch, but also among pericytes on disparate capillaries. Our analysis indicates that this arrangement was not a result of inherent isotropy of the vascular network itself, but rather is evidence for the optimized activity sensing across the retinal tissue. However, such a “symmetric” structure poses a challenge: how to discriminate an active region from its quiescent neighbor? We showed that the spatial accuracy of the vasomotor response was mediated by intricate connections among vascular cells along the feeding vascular branch. We found that pericytes and ECs form a functional relay unit by coupling to each other, and not to surrounding neurons and glia. This is intriguing given the abundant expression of various connexins across a wide range of cells in the retina^13^. While the regulatory mechanisms for this GJ-mediated connectivity remain unclear, this cellular specificity may provide an important insight into the nature of the vascular signaling and its accuracy. The limited coupling between SMCs of the arterioles that we demonstrate here, in concert with the presence of a vascular sphincter^36,37^, a specialized structure at the pre-capillary region, may have implications to limiting the spread of vasoactive signal past the activity site, a necessary step to avoid a non-specific broad blood supply. Thus, the spatially contained vasoactive signaling during functional hyperemia supports the presence of local functional domains^8^.

### Spatial tuning of neurovascular coupling

Our findings also suggest that GJ-mediated connectivity is not only capable of spatially precise delivery of blood to the active region, but is optimized to focal stimulation. The strengthening of both vascular cell coupling and vasomotor response during focal stimulation is likely driven by an optimized neuronal response during the receptive field center stimulation. This finding is significant as it establishes the resolution of fMRI BOLD imaging at the level matching the receptive field of neuronal interactions. It also provides a mechanistic basis for in vivo observations of a spatial resolution at 100–400 µm, corresponding to the areas of cortex, activated by a whisker stimulation or by a single ocular dominance column in the visual cortex^38–41^. Such spatial precision of vasomotor response would require the recruitment of the contractile cells exclusively along the active vascular branch and only up to the active site. We found that the recruitment of contractile cells along the active branch was mediated through the discriminative coupling of the vascular cells. This GJ-mediated coupling was essential for the fast propagation of a Ca^2+^ wave leading to a vasomotor response along the vascular branch. Ca^2+^ wave was restricted to the vascular cells without involvement of neurons, astrocytes, or Müller cells, consistent with a recently described vascular relay circuit^13^. It was likely spreading through ECs, as the current spread through the microvascular endothelium has much higher efficiency than between pericyte-to-pericyte and pericyte-to-EC^14,16,42,43^. This fast and non-decaying signal propagation through ECs may rely on active regenerative mechanism^44^ and allowed nearly instantaneous activation of cells along the active vascular branch. Interestingly, we showed that both Ca^2+^ dynamics and excitation-contraction coupling was most efficient in SMA, which may explain faster vasomotor response on precapillary regions than in capillaries, as shown both in vitro^42^ and in vivo^8^.

Once the appropriate vascular branch has been activated, the next task is to restrict signal propagation to the active branch without affecting other areas that are supplied by the same feeding artery. First, our cellular tracing and GCaMP6f experiments using intact retina revealed that vascular cells were electrotonically insulated from the surrounding glia and neurons, thus limiting radial spread. Second, pericytes and ECs coupled strongly along with the capillary and weakly to ECs and SMCs on the arteriole. This is consistent with the earlier electrotonic studies in isolated vascular branches where the conductance dropped abruptly at the branching point^14^. Extending recent reports, we also showed that the vasomotor signal was observed from the upstream arteriole up to the active side^8,16,45^. Consistently, vascular cell coupling, Ca^2+^ wave and vasomotor responses were recorded on the upstream side of the active region. This directional response toward the feeding artery was driven exclusively by local NO production and was lost during global sensory stimulation or bath NO application. We further hypothesized that this NO-mediated directionality depended on the preferential opening of ECs’ GJs in the upstream direction. ECs are highly polarized with respect to blood flow^46–48^. Specifically, caveolae with caveolin-1 and eNOS are abundant in the upstream end of ECs^49^. Caveolin-1 inhibits the activity of eNOS. Upon shear stress^50^ or Ca^2+^ elevation (as in our zap experiments), eNOS leaves caveolin, binds to Ca^2+^-calmodulin and produces NO^51^. Thus, NO production appears to be compartmentalized^52^ in the upstream end of ECs. Consistent with our experiments, NO was shown to nitrosylate Cx43 GJs resulting in their opening^53,54^. While further studies are needed, polarized distribution of caveolin-1-eNOS complex in EC may be a mechanism to control directionality and spatial precision of vasomotor response via selective activation of GJs.

### Implication to neurovascular pathology in diabetic retinopathy

In diabetes, vascular pathology is associated with high glucose, causing activation of protein kinase C pathway^55–57^ and reduced activity of Cx43^6,12,58–60^. In our experiments with the retina of diabetic mice, cellular coupling, vasomotor response, and blood flow were impaired. This is consistent with reduced flicker response and abnormal blood flow in the retina of diabetic patients^60–62^. As we showed in a mouse model of diabetic retinopathy, expression of the Cx43 GJ along the vascular relay is preferentially downregulated^6,13^. Downregulation of GJs leads to restriction of Ca^2+^ wave and vasomotor response experiments along the vascular branch, consistent with a ~5-fold increase in voltage decays along the retinal microvasculature in rat model^63^. Disrupted vascular cell connectivity and reduced responses to vasoactive signals may contribute to declining functional hyperemia observed early in the disease.

## Material and methods

In all experimental procedures, animals were treated in compliance with protocols approved by the Institutional Animal Care and Use Committee (IACUC) of Weill Cornell Medicine (WCM), and in accordance with the National Institutes of Health Guide for the Care and Use of Laboratory Animals. The use and application of STZ were in accordance with safety protocols approved by WCM’s Environmental Health and Safety (EHS), Institutional Biosafety Committee (IBC) and IACUC Protection and Control sub-committee (P&C).

### Experimental animals

Diabetes was induced in three mouse lines: C57BL/6 mice (Jackson Laboratory, Stock#: 000664, RRID:IMSR_JAX:000664), NG2-DsRed mice (Jackson Laboratory, Tg(Cspg4-DsRed.T1)1Akik/J, Stock#: 008241, RRID:IMSR_JAX:008241) and NG2-Cre-GCamp6f, generated by crossing FVB-lfi208Tg(Cspg4-cre)1Akik/J (Jackson Laboratory, Stock#: 008533, RRID:IMSR_JAX 008533) with Ai95(RCL-GCaMP6f)-D loxP (Jackson Laboratory, Stock#: 024195, RRID:IMSR_JAX 024195). Breeding pairs negative for the *rd1* mutation were used to produce animals for this study. We used the STZ diabetic mouse model^57^. Male mice aged 6–8 weeks were fasted for 4 h prior to the injections. The animals were injected intraperitoneally on 5 consecutive days with 50 mg/kg STZ (Sigma-Aldrich, S0130) freshly dissolved in a citrate buffer (pH 4.5). Control animals received a citrate buffer injection without STZ. In our STZ mouse model of diabetes, the levels of blood glucose reached maximum elevation 1 month after STZ injection and remained elevated. The diabetes was defined by non-fasting blood glucose >300 mg/dL verified 1 month after the last STZ injection and confirmed on the day of the experiment.

### Retina wholemount preparation

Methods for wholemount tissue preparation have been described in detail previously^58^. After the animal was euthanized, its eyes were enucleated and placed in bicarbonate-buffered Ames solution (Ames; Sigma, A1420), equilibrated to pH 7.4. It has been shown that variations in the O_2_ level in brain tissue can affect functional hyperemia^5^. To reduce any discrepancy, O_2_ level was maintained between 19% and 24%, checked with an oximeter (WPI ISO2-D) in the chamber. After dissection of the eyes, cornea, iris, and lens were removed. The retina was dissected into four equal quadrants and attached photoreceptor surface down on a modified Biopore Millicell filter (Millipore). This preparation was transferred to a recording chamber and bathed (1 ml/min) with Ames. Pharmacological agents were also prepared in Ames. All experiments were performed at a near physiological temperature of 32 °C.

### Identification of pericytes, mosaic measurements, and conformity ratio

In the retina wholemount, identified pericytes were targeted on capillaries in the superficial vascular layer, avoiding those located on arterioles and veins. Capillaries were defined based on several morphological criteria: (1) diameter not exceeding 10 μm, approximately equivalent to the diameter of red blood cells, which are readily present in the living tissue, (2) lack of smooth muscle actin. Initially, we targeted fluorescently labeled pericytes in NG2-DsRed mice. In genetically unmodified mice, we identified pericytes based on “bump on a log” appearance of the individual pericytes on the abluminal side of the vessel wall^64^, or by using 10-min incubation in NeuroTrace 500/525 Green Fluorescent Nissl Stain (1:200 in HEPES-Ringer)^20,32^. We measured the NND and the SRAV between pericytes using ImageJ (NIH, USA). NND was defined in an arrangement of collapsed optical slices within each vascular lamina. We measured *n* = 275 NNDs between pericyte somas on the collapsed ImageJ, simple neurite tracer in a 3D reconstruction. We made *n* = 141 measurements from all three layers. The SRAV routes were manually checked for errors, less than 2% had to be corrected (e.g., manually link non-touching pericyte-endfeet). Normal distribution of datasets for mosaic arrangement had been tested with Excel descriptive statistics kurtosis (<1) and skewness (<1). The conformity ratio (CR), a commonly used quantitative measure of mosaic regularity, was calculated as a ratio of the mean inter-pericyte distance to the corresponding standard deviation^21^.

### Cellular coupling and directionality assessment

Cell coupling was assessed using a NB probe^13^. The use of NB was justified by the following considerations. Due to its small size (286 Da), NB easily permeates the smallest GJs^65^. Since most GJs are preferentially permeable to cations^66^, a cationic nature of NB allows for a better diffusion, which is further boosted by the electroporation. In contrast to Lucifer Yellow or other tracers, NB has low cellular toxicity. It is fixable and compatible with additional antibody labeling, necessary for the identification of coupled cells. The visualization of NB with Streptavidin allows additional amplification of the NB signal to reveal the weakest connections between the coupled cells.

For cellular injections, one mid-periphery cell per retina quadrant was targeted. Injection pipettes were pulled from borosilicate glass (1B150F-4) with a P-97 Flaming/Brown puller and had a resistance of ∼1–2 MΩ. First, the pipette was filled with filtered Ames solution supplemented with 100 μg/mL papain (~1 Unit/mL). To dissolve the vascular basement membrane covering pericytes, the papain-containing solution was focally applied around the target cell for 5 min. Second, a fresh pipette was filled with intracellular solution containing: 120 mM Cs-gluconate, 10 mM tetraethylammonium chloride (TEA-Cl), 1.0 mM CaCl_2_, 1.0 mM MgCl_2_, 11 mM ethylene glycol-bis(β-aminoethyl ether)-N,N,N′,N′- tetraacetic acid (EGTA), and 10 mM sodium N-2-hydroxyethylpiperazine-N′-2-ethanesulfonic acid (Na-HEPES), adjusted to pH 7.2 with CsOH. The solution was supplemented with 2% NB (Vector, SP-1120) and 0.5% Alexa488-hydrazide (Thermo Fischer Scientific, A10436). This new pipette was pressed against the cleaned target and the cell membrane was gently pulled inside the pipette until a >200 MOhm seal was established. The alternating voltage steps between –300 mV and +50 mV, 2 Hz, were applied for 1 min to confirm successful targeting of a cell after filling with Alexa488-hydrazide. If the target cell was selectively backfilled with Alexa488, NB was electroporated for additional 3 min using +200 mV –50 mV, 2 Hz voltage steps. If either the targeted cell was not filled with Alexa488 or Alexa was detected outside of the injected cell, the preparation was discarded. Following NB electroporation, the preparation was left for 15 min to allow intercellular NB diffusion. All electroporations were made with a MultiClamp 700B patch-clamp amplifier (Molecular Devices, Sunnyvale, CA, USA) using Signal software (CED, UK). For detection with 20× objective, up to all three (SL, IL, and DL) retinal vascular layers were imaged and up to the depth of visible spreading with high digital amplification (above background) for 60×, oil objective to detect all possible NB^+^ cells during 60×, high-resolution rescans. When the hyperstacks were processed, all images were intensity normalized and reconstructed in 3D with FIJI (ImageJ). We used IsoData thresholding for defining positive cells in FIJI. The thresholded images were then Z-merged up to the depth of positive cells, or up to the depth of the retinal vascular layer (indicated locally). To produce 3D rotations, we used interpolated 3D Project in the desired X or Y angle.

For each targeted cell type, a coupling strength (CS) was measured as a ratio of the NB stain fluorescence intensity in cell bodies of six nearest neighbors to the NB stain intensity in the injected cell body. Therefore, the values approaching 1 indicate strong coupling, while 0, such as in the presence of GJ blocker MFA, no coupling (Fig. 2f). In case of pericytes and ECs, NB stain intensity was measured in three cells in each direction up- and downstream of the vascular branch from the injected cell (Fig. 2e). When heterocellular coupling was detected, the CS was measured for each cell type combination to yield a coupling matrix (Fig. 2g). The directionality of vascular cell coupling was quantified using a directionality index (DI), a ratio between NB pixel intensity within the upstream vascular branch to downstream vascular branch from the injection point. DI values >1 indicate a connectivity bias toward the feeding artery. This simple approach provided a robust and reproducible assessment of the response directionality bias and was not intended to discriminate between pericytes and EC involvement.

### Vasomotor response induction and quantification

Pericytes were focally stimulated under an upright Nikon FN1 microscope by a current pulse (7 μA, 2 ms; Grass Technologies) using an electrode filled with Ames solution. Electrodes were pulled from borosilicate glass (WPI, 1B150F-4) with a P-97 Flaming/Brown puller (Sutter Instruments, Novato, CA, USA) and had a measured resistance of 3–5 MΩ. For consistency across all experiments, the electrode was placed near the cell body of the targeted pericyte. During focal “puff” stimulation, the electrode solution was supplemented with a vasoactive compound and delivered with picospritzer (Parker Hannifin) via a broken patch pipette positioned above the targeted pericyte. For the light stimulation experiments, the microscope’s illuminator was used to deliver a spot of light that was centered on the targeted pericyte cell body and focused on the photoreceptor cell layer. The tissue was adapted at 30 cd/m^2^, and the stimulus was 270 cd/m^2^. Light spot flicker (40 µm diameter, 10 Hz) was controlled by a shutter (Uniblitz, Vincent Associates). Responses to stimuli were captured on video or time-lapse photos with a microscope-mounted Sony A7s full-frame camera. Images of blood vessels were analyzed in ImageJ, using a region of interest (ROI) tracing tool. At each experimental condition, the capillary lumen cross-sections were mapped at 2 μm steps along the capillary.

### Two-photon assessment of calcium dynamics, vasomotor response and blood flow

Retinal quadrants attached to a filter were transferred to a recording chamber on the stage of an upright ThorLabs Bergamo II two-photon microscope with a tunable femtosecond TI sapphire laser. Retinal quadrants were attached on the filter and bathed in flowing at 1 mL/min bicarbonate-buffered Ames solution (Ames; Sigma, A1420), equilibrated to pH 7.4 at 32–33 °C. The solution was continuously bubbled with carbogen. GCamp6f, Evans Blue, and DsRed were simultaneously excited at 920 nm and the emission was split into corresponding channels, 520 nm for GCaMP6f, 580 nm for DsRed, and 680 nm for Evans Blue. Evans Blue was used to visualize blood vessels and was intraperitoneally injected in the living mouse 30 min prior to dissection^30^. Identified pericytes were targeted on capillaries in the superficial vascular layer. In the initial experiments in NG2-DsRed non-diabetic mice, we targeted fluorescently labeled pericytes. In genetically unmodified mice, we were able to identify pericytes in contrast optics based on “bump on a log” appearance of the individual pericytes on the abluminal side of the vessel wall^64^. Pericytes on straight capillaries and at the forks were focally stimulated by a 10–20-μA and 2 ms current pulse (Grass Technologies) using an electrode filled with HEPES-buffered extracellular Ringer’s solution, containing the following: 137 mM NaCl, 2.5 mM KCl, 2.5 mM CaCl_2_, 1.0 mM MgCl_2_, 10 mM Na-HEPES, 28 mM glucose, pH 7.4. Electrodes were pulled from borosilicate glass (WPI, 1B150F-4) with a P-97 Flaming/Brown puller (Sutter Instruments) and had a measured resistance of ∼5 mΩ. For consistency across all experiments, the electrode was inserted under the limiting membrane and placed near the cell body of the targeted pericyte. Videos were taken at 15 fps rate. All data were analyzed in ImageJ. In all images, the background was removed and the GCaMP6f signal was measured in regions of interest as changes to baseline ratio (∆*F*/*F*). Time of 10% and 90% Ca^2+^ increase was calculated from the moment of electric stimulation to 10% or 90% increase of ∆*F*/*F* Ca signal, correspondingly. For analysis of vasomotor response, Evans Blue labeling was thresholded and vascular diameter was automatically estimated for each frame using the line profile algorithm in ImageJ. The vasomotor response was calculated in Microsoft Excel as changes to the baseline ratio (∆*D*/*D*). Times to reach 10% and 90% vasoconstriction were calculated from the moment of electric stimulation.

For the blood flow assessment in vivo, intraperitoneal injection of 100 μL (100 μg/mL) Evans Blue was done 30 min prior to measurements. The animal was anesthetized with a mixture of 150 mg/kg ketamine and 15 mg/kg xylazine. The pupils were dilated with 0.5% tropicamide ophthalmic solution, and a coverslip was placed on each eye with GONAK ophthalmic solution. Mice were mounted with SG-4N mouse head holder (Narishige) on an upright ThorLabs Bergamo II two-photon microscope. Blood flow was measured under a 10× super apochromatic objective with a 7.77-mm working distance and 0.5 NA (TL10X-2P, ThorLabs, Newton, NJ, USA). Evans Blue was illuminated with 920 nm wavelength and the measurements were taken at 116–400 frames per second rate. Baseline blood flow in control non-diabetic and STZ-treated mice were recorded. Pre-diabetic animals were 2–3 weeks post last STZ injection and their non-fasting glucose was below 250 mg/dL. Diabetic animals were used 3–4 months after the last STZ injection and their glucose was consistently above 300 mg/dL. Blood flow was estimated as the number of blood cells passing through a capillary per second. This analysis was performed in ImageJ by plotting fluorescence profiles across the blood vessels at every frame. In the resulting plot, the downward peaks indicated passage of a blood cell, which was darker relative to the labeled plasma. The peaks were automatically detected in Microsoft Excel and verified by visual inspection of the original data^32^.

### Immunohistochemistry

After vasomotor assessment and/or pericyte tracing each sample, still attached to the Biopore insert, was submersion-fixed in freshly prepared fixative (0.25% PFA, 4% carbodiimide in Phosphate-buffered saline (PBS)) for 15 min at room temperature. The fixed samples were washed in PBS and the retinas were separated from the insert. We visualized NB overnight with streptavidin-A488, mixed with To-Pro-3 Iodine (1:30,000; far red, T3605, Thermo Fisher Scientific). In multi-labeling experiments, wholemounts were incubated in a mixture of primary antibodies, followed by a mixture of secondary antibodies. Retina wholemounts were blocked for 10 h in PBS, containing 5% Chemiblocker (Chemicon), 0.5% Triton X-100, and 0.05% sodium azide (Sigma, St. Louis, MO, USA). Primary antibodies were diluted in the same solution and applied for 72 h, followed by incubation for 48 h in the appropriate secondary antibody, conjugated to Alexa488 (1:1000; green fluorescence, Molecular Probes), Alexa568 (1:1000; red fluorescence, Molecular Probes), or Cy5 (1:500; far-red fluorescence, Jackson). All steps were carried out at room temperature. After staining, the retinal pieces were flat mounted on a slide, ganglion cell layer up, and coverslipped using Vectashield mounting medium (H-1000, Vector Laboratories). The coverslip was sealed in place with nail polish. To avoid extensive squeezing and damage to the retina, small pieces of a broken glass coverslip (number 1 size) were placed between the slide and the coverslip. The primary antibodies used in this study were the following: rabbit anti-Cx43 (Cx43, 1:2000, Sigma-Aldrich, C6219, RRID:AB_476857), rabbit anti-NG2 coupled to Cy3 fluorescent label (NG2, 1:500, EMD Millipore, AB5320C3, RRID:AB_11214368). Retinal samples were imaged under a Nikon Eclipse Ti-U confocal microscope (Morell Inst., Melville, NY, USA). The samples were imaged under identical acquisition conditions, including: laser intensity, photomultiplier amplification, and Z-stack step size. All images were processed and analyzed using ImageJ (for details see related sections). In total, we evaluated 1007 individual pericytes in non-diabetic and 561 pericytes in diabetic animals.

### Statistical analysis

Statistical analysis was performed in SigmaPlot 14 (Systat, RRID:SCR_003210). For multiple comparisons, analysis of variance (ANOVA) with Tukey’s post-hoc, or repeated measures ANOVA were used. The data are presented as means ± SD. *n* is the number of animals per group. To avoid the introduction of non-independent data into statistical analysis, first, multiple samples were averaged within the animal, then the data between animals were compared^67^.

## Supplementary information

Supplementary information

Supplementary Video S1

Supplementary Video S2

Supplementary Video S3

## Supplementary information

**Supplementary Information** accompanies the paper at (10.1038/s41421-020-0180-0).
