# Supplementary material for: The pericyte connectome: spatial precision of neurovascular coupling is driven by selective connectivity maps of pericytes and endothelial cells and is disrupted in diabetes
Source: Cell Discov. 2020 Jun 16;6:39. doi: 10.1038/s41421-020-0180-0 (PMC7296038; doi:10.1038/s41421-020-0180-0)
Supplement: Supplementary file 1 — Supplementary information [file 41421_2020_180_MOESM1_ESM.pdf]

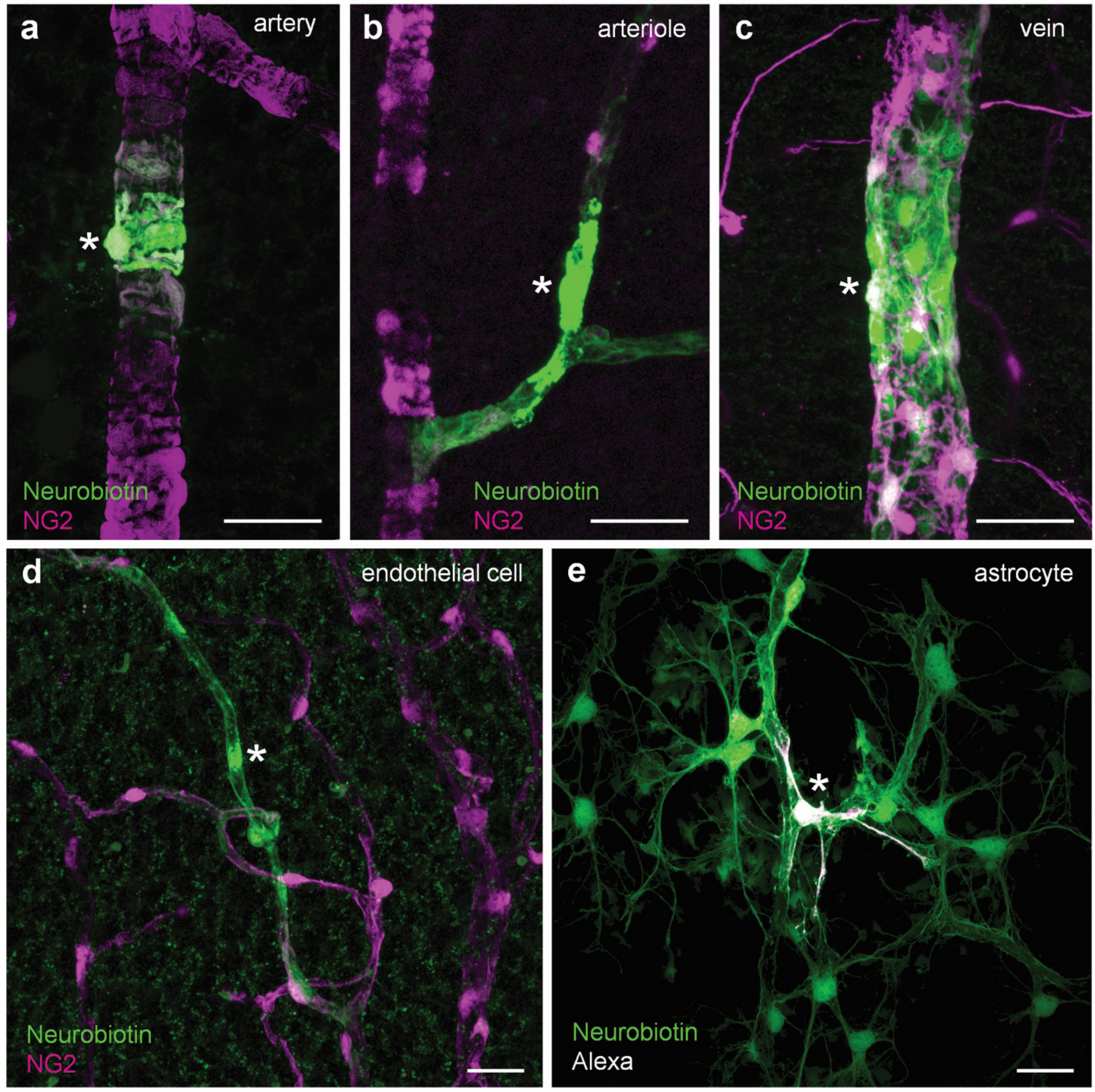

Supplementary Fig. S1 Gap junction-mediated coupling patterns across smooth muscle cells, pericytes, endothelial cells and astroglia. a, b Representative tracing results following arterial **(a)** and arteriolar **(b)** terminal smooth muscle cell injection with Neurobiotin probe (green) in the NG2-DsRed mouse retina (magenta). **c** Mural cell coupling pattern in the retinal vein. **d**, **e** Representative tracing results following targeted Neurobiotin injection into endothelial **(d)** and astroglial **(e)** cells. * **-** injected cell. Scale bar: 50 μm.

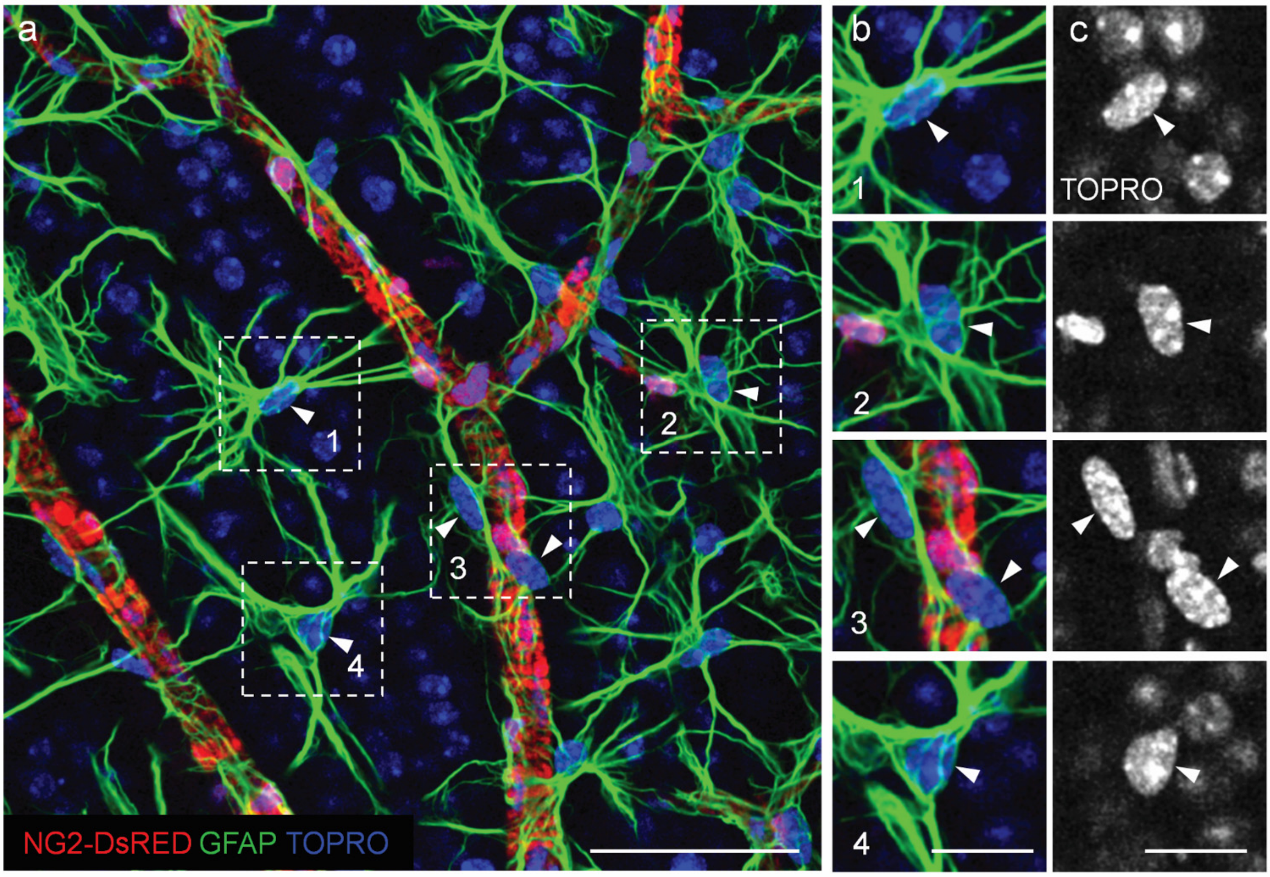

Supplementary Fig. S2 Identification of astroglia by a nuclear morphology. a Confocal image of an NG2-DsRed retina wholemount co-stained for astroglia-specific marker GFAP (green) and ubiquitous nuclear marker TOPRO (blue). **b** High magnification confocal images from the corresponding regions from **a**, showing a distinct nuclear morphology of identified astroglia. **c** Astroglia nuclei (arrowhead) appear large, elongated and dense, making them easy to distinguish. Scale bars: 50 µm (**a**), 10 µm (**b, c**).

Supplementary Video S1 3-D reconstruction of pericyte GJ-mediated connections across all vascular layers in the retina of NG2DsRed mice.

Supplementary Video S2 *In vivo* 2-P imaging of capillary blood flow in the nondiabetic retina (1:10 speed).

Supplementary Video S3 *In vivo* 2-P imaging of capillary blood flow in the diabetic retina (1:10 speed).
